# Knowing How You Know: Toddlers Reevaluate Words Learned From an Unreliable Speaker

**DOI:** 10.1162/opmi_a_00038

**Published:** 2021-02-01

**Authors:** Isabelle Dautriche, Louise Goupil, Kenny Smith, Hugh Rabagliati

**Affiliations:** Laboratoire de Psychologie Cognitive, Aix-Marseille University, CNRS, Marseille, France; Institute of Language, Communication and the Brain, Aix-Marseille University, CNRS, Aix-en-Provence, France; School of Psychology, University of East London, London, UK; University of Edinburgh, Edinburgh, UK; University of Edinburgh, Edinburgh, UK

**Keywords:** source monitoring, word learning, selective trust, metacognition

## Abstract

There has been little investigation of the way source monitoring, the ability to track the source of one’s knowledge, may be involved in lexical acquisition. In two experiments, we tested whether toddlers (mean age 30 months) can monitor the source of their lexical knowledge and reevaluate their implicit belief about a word mapping when this source is proven to be unreliable. Experiment 1 replicated previous research (Koenig & Woodward, 2010): children displayed better performance in a word learning test when they learned words from a speaker who has previously revealed themself as reliable (correctly labeling familiar objects) as opposed to an unreliable labeler (incorrectly labeling familiar objects). Experiment 2 then provided the critical test for source monitoring: children first learned novel words from a speaker before watching that speaker labeling familiar objects correctly or incorrectly. Children who were exposed to the reliable speaker were significantly more likely to endorse the word mappings taught by the speaker than children who were exposed to a speaker who they later discovered was an unreliable labeler. Thus, young children can reevaluate recently learned word mappings upon discovering that the source of their knowledge is unreliable. This suggests that children can monitor the source of their knowledge in order to decide whether that knowledge is justified, even at an age where they are not credited with the ability to verbally report how they have come to know what they know.

## INTRODUCTION

Children learn words through iterative social interactions. For example, in order to determine the meaning of the word “dog,” English children may repeatedly observe other people using that word until, across these situations, its referent and meaning became clear. Research on cross-situational learning suggests that children have a fine-grained sensitivity to patterns of association that exist between words and the world, which they can use to gradually update their knowledge of a word’s meaning (e.g., Colunga & Smith, 2005; Regier, 2005; Siskind, 1996; L. Smith & Yu, 2008; L. B. Smith, 2000). But much less is known regarding how this updating process is affected by social factors, such as the reliability of their source. Here, we ask whether toddlers between 2 and 3 years of age can also update their knowledge of how they came to know a word’s meaning, and use that information when constructing and updating their lexicon.

Being able to reason about the sources of one’s own knowledge is fundamental for domains where learners depend on others to gain knowledge, such as in acquiring a lexicon, because the continuous flow of social interactions will often require learners to dynamically update previously acquired knowledge, based upon newly learned properties of their partners. For example, when learning words, conversational partners may appear to be good models at first, but later reveal themselves to lack important knowledge, or be from another community (perhaps with different naming conventions), or even be untrustworthy. In cases like these it would thus be important for the learner to reconsider those initially learned words, and potentially expunge them from their lexicon. Sperber et al. (2010) have argued that humans have evolved a suite of mechanisms for this “epistemic vigilance,” which let us scrutinize communicated information for its veracity through assessing both its content and its source. One may thus predict that these mechanisms would play a crucial role for stabilizing linguistic forms within a community, because they minimize the risk of being (mis)informed by unreliable sources. For children in the process of learning language, tracking how they have come to know a word’s meaning and scrutinizing informants for their competence with a particular language may constitute an important mechanism for minimizing “errors” in the lexicon, in the face of uncertain and occasionally misleading social interactions.

Much research has documented that children, at least in their preschool years, are not without resources when it comes to selecting informants (Harris, 2015; Poulin-Dubois & Brosseau-Liard, 2016). For example, by 4 to 5 years of age, children prefer to learn from more reliable speakers (e.g., Corriveau et al., 2009; Koenig et al., 2004; Koenig & Harris, 2005; Pasquini et al., 2007). In these studies, children first gathered information from and about two speakers, such as witnessing reliable and unreliable speakers labeling objects differently (e.g., calling a ball either “ball” or “dog,” respectively). In subsequent novel word learning tasks, children preferentially endorsed the label used by the reliable speaker over the one used by the unreliable speaker, at least by the age of 4. In and of itself, this result could be explained through fairly simple associative mechanisms, whereby children generalize inaccuracy from a speaker’s past behavior to a current label. But other studies show that children’s inferences are based on a more sophisticated understanding, such that they are not merely tracking the surface accuracy of each speaker but are instead making inferences about the causes of each speakers’ behavior to inform their reliability judgment (Einav & Robinson, 2011; Nurmsoo & Robinson, 2009). For instance, Nurmsoo and Robinson (2009) show that preschoolers do not infer that speakers are unreliable if they can explain away inaccurate behavior because of situational factors (e.g., being blindfolded) rather than epistemic states.

Critically, 4- to 5-year-olds are not only able to use trustworthiness information for subsequent learning, they can also reevaluate what they have learned about a word when *later* discovering that a speaker is unreliable (Luchkina et al., 2020; Schütte et al., 2019; Scofield & Behrend, 2008). In these experiments, children first learnt novel words from a speaker and only later discovered that the speaker was either reliable or unreliable (i.e., by witnessing them labeling known objects correctly or incorrectly). Subsequently, the children showed no recognition of the form-meaning mapping taught by the unreliable speaker, while remembering the mapping taught by the reliable speaker, which indicates that after initially learning the words, they subsequently used the speaker reliability information to reevaluate the mappings, providing evidence that on top of tracking the source of their knowledge, they can also reflect on it to reevaluate the likely accuracy of their knowledge.

While reasoning about knowledge sources seem to be well in place in the preschool years, a critical question is whether such a mechanism is in place during the earliest stages of lexical development, helping children to filter the information communicated to them, in order to learn words more rapidly and optimally. Classic work suggests that children younger than four struggle to explicitly identify the source of their knowledge (Gopnik & Graf, 1988; Lindsay et al., 1991; O’Neill & Gopnik, 1991; Taylor et al., 1994), but more recent evidence suggests that even toddlers can evaluate the trustworthiness of a speaker in order to guide subsequent word learning (Brooker & Poulin-Dubois, 2013; Crivello et al., 2018; Koenig & Woodward, 2010; Luchkina et al., 2018). For instance, in Koenig and Woodward (2010), 24-month-olds interacted with an accurate (labeling familiar objects correctly) or inaccurate (using wrong labels) speaker who taught them a novel word-object mapping. When a second speaker requested the target object from them, only children who were previously exposed to the accurate source showed above chance performance in understanding the novel word, suggesting that children do not generalize novel words taught by inaccurate speakers to other speakers. However, it is still unclear whether these demonstrations of early-developing selectivity in learning are driven by simple associative mechanisms, whereby toddlers would simply ignore the information delivered by speakers who previously revealed themselves to be unreliable, or by higher order social processes whereby children appraise and reason about the reliability of their sources, and this is the subject of an ongoing debate (Crivello & Poulin-Dubois, 2019; Heyes, 2017). To date, the only relevant evidence is correlational: toddlers’ selective learning in those tasks is related to their metacognitive and mindreading skills, but not to their associative learning skills (Crivello & Poulin-Dubois, 2019; Kuzyk et al., 2019). These correlations could potentially support a rich interpretation of infant’s selective learning behavior, but causal (rather than correlational) evidence is required.

In the present study, we provided a more direct test of toddlers’ ability to reason about the source of their linguistic knowledge. Our method differs from prior studies, which focus on whether children *filter out* information coming from an unreliable source. Rather, we ask whether toddlers will *reevaluate* knowledge learned from a source upon receiving new information about her reliability. If they do, this would suggest that, years before being credited with the ability to verbally report upon the source of their knowledge, young children can still reason about the source of their knowledge and use this information to reevaluate previously acquired information.

Experiment 1 aimed to replicate previous findings that 2-year-olds use evaluations of trustworthiness to guide word learning, and thus learn novel words from a reliable speaker but not from an unreliable one. To reduce the task demands for these younger children, we used a between-participant design where one group of children is presented with a reliable speaker and the other group with the unreliable speaker (see Brooker & Poulin-Dubois, 2013; Koenig & Woodward, 2010) and we tested children’s knowledge of the novel words using a preferential looking task, using eye-gaze as an implicit correlate of children’s knowledge (see also Luchkina et al., 2018). In this experiment, children were first exposed to a speaker that provided either correct labels for familiar objects (e.g., saying “ball” while playing with a ball; the reliable speaker) or incorrect labels (e.g., saying “dog” while playing with the same ball; the unreliable speaker) before the speaker taught them two novel labels for two novel objects. The test phase (identical across speaker conditions) was then administered by another reliable speaker. Following prior work, we only expected children to learn new words from the reliable speaker.

Experiment 2 then provided the critical test of reevaluation: We used the same procedure, except that this time children were taught the novel words before being given the chance to observe the speaker’s reliability. We relied on the result that children presuppose a generally truthful use of speech (Corriveau et al., 2009; Jaswal & Neely, 2006) and thus, by default, should initially have learned the meanings of the novel words in both conditions. Results from word learning studies using the same, or similar, teaching procedure support this assumption as they show that toddlers of this age successfully learn novel words taught on screen by a speaker they have no experience with (Dautriche et al., 2015; Swingley & Aslin, 2007; Waxman & Booth, 2002). We thus tested whether children were able to retrospectively reevaluate their knowledge of the novel word when presented with evidence that the speaker who taught them the word was unreliable.

## EXPERIMENT 1

### Method

The preregistration, the data, and the script for their analysis are available here: https://tinyurl.com/y2w8ymmy. We note below when our analyses departed from the preregistration.

#### Participants.

Forty-eight English-speaking children ranging from 24 months to 36 months took part in this experiment (*n* = 24 in each condition; reliable condition: *M* = 29*M*, 20*D*, *SD* = 121*D*, 10 boys; unreliable condition: *M* = 30*M*, 16*D*, *SD* = 102*D*, 12 boys). The sample size was determined based on Koenig and Woodward (2010) who tested 20 participants in each condition in a similar design (albeit with a different measure; Cohen’s *d* = 0.8). A power analysis based on this effect suggested that we should at least test 24 children per group to have a power of 80% at the .05 alpha level. Four additional children were replaced because of fussiness during the experiment resulting in the absence of calibration (*n* = 1), noise in the experimental settings requiring the experimenter to play the experimental material twice (*n* = 2), or because English was not the dominant language (*n* = 1). Participants were recruited in nurseries around Edinburgh (*n* = 37) and in the lab (*n* = 11).

#### Procedure, Design and Material.

**Figure 1.**
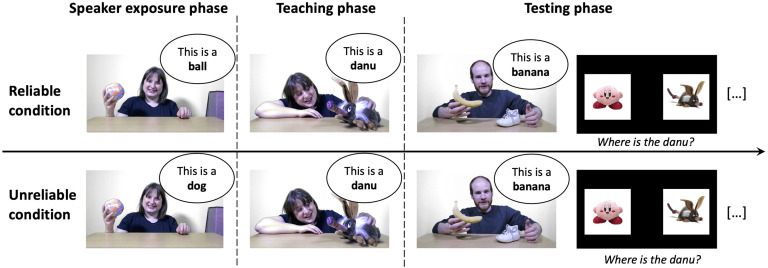
**Design of Experiment 1.** The experiment consisted of three phases: the speaker exposure phase where a speaker was labeling familiar objects, the teaching phase where the speaker was teaching two novel words (“danu” and “modi”), and the testing phase, which included included a short video of a second reliable speaker and a succession of 16 trials: eight test trials (as pictured, with the two novel objects on the screen) and eight familiar trials (with two known objects on the screen). The critical difference between the conditions happened during the speaker exposure face: In the reliable condition, the speaker used the correct word to label the object she was playing with (e.g., calling a ball “ball”), however in the unreliable condition, the speaker used the wrong label (e.g., calling a ball “dog”).

Children were either tested in their nursery or in the lab. They sat on a small chair in front of a laptop with the experimenter sitting next to them. The experimenter greeted the child before introducing them to a game (the experiment). The accuracy of the speaker was not mentioned during the experiment. The experimenter avoided responding to any-task relevant comments the child might have said. The experiment was composed of three phases (see Figure 1):

1.  **Speaker exposure phase.** Participants saw a video of a native English female speaker playing with five objects and labeling them. Each object was taken out of a box individually, labeled three times and put back into the box. Half of the participants heard the speaker using the correct label for the object she was playing with (the *reliable condition*; e.g., “This is a ball! Look, a ball! This ball is really nice” while playing with a ball) and the other half heard the speaker using an incorrect label (the *unreliable condition*; e.g., “This is a dog! Look, a dog! This dog is really nice” while playing with the ball). The same five objects were used across the two conditions: a tiger puppet, a banana, a ball, a shoe, and glasses. In the unreliable condition, the speaker used labels that referred to objects that did not appear anywhere else in the experiment (i.e., “flower,” “car,” “dog,” “book,” “star”).
2.  **Teaching phase.** Next, participants watched two videos, each teaching them one novel word. Each video was about 30 s long and showed the speaker seen during Phase 1. In each video the speaker showed a novel object and labeled it five times using one of of two novel words (“danu” or “modi”).
3.  **Testing phase.** The test phase assessed children’s learning and generalization of the names. We used a second reliable speaker for these trials, to minimize the possibility that children would treat the unreliable labels as situationally defined (see Koenig & Woodward, 2010): that is, relevant for communicating with the unreliable speaker, and thus recognizable if tested with that speaker.^1^ We chose a male speaker to maximize children’s ability to differentiate the voice of the novel speaker during the test phase from the voice of the female speaker seen in Phase 1 and 2. To ensure that children would recognize the novel speaker as reliable, participants first saw a short 15-s video of the speaker. The speaker greeted the child and placed two familiar objects in front of him, a shoe and a banana. He then asked the child whether (s)he knew where the banana is (“Do you see the banana?”) and then picked the banana after a brief delay (“Here it is! Here is the banana!”).

After watching the video, participants were tested using a preferential looking procedure, in which they saw two objects on screen and heard the novel speaker name one of the objects. We chose looking time as a measure to minimize task demands, which has been successfully used in word learning paradigms at this age range (e.g., Messenger & Fisher, 2018; Naigles, 1990; Yuan & Fisher, 2009). Participants were administrated a total of 16 trials: eight familiar word trials and eight novel word trials, four per novel word. Each trial started with the simultaneous presentation of two pictures on the right and left sides of the screen (the baseline period). Two s later, the test sentences started, for example, “Look at the [target]! Do you see the [target]?” The target word was pronounced twice in each trial. The trial ended 4 s after the first target word onset. Targets appeared eight times on the right side of the screen and eight times on the left side of the screen across the testing phase, and the target was not on the same side on more than two consecutive trials. Importantly, the audio stimuli was recorded by the reliable male speaker.

#### Materials.

##### *Exposure phase*.

The five labels used during the exposure phase in the reliable condition (“tiger,” “banana,” “ball,” “shoe,” “glasses”) and in the unreliable condition (“flower,” “car,” “dog,” “book,” “star”) were chosen such that they are all likely to be known by children of that age range. According to Wordbank (Frank et al., 2017), 87% of British 24-month-olds understand the words used in the reliable condition and 94% in the unreliable condition. Based on parental report (on the 35 questionnaires that could be collected, 17 from children assigned to the reliable condition and 18 from children assigned to the unreliable condition), participants in the reliable condition knew on average 97% (*SD* = 6.6%) of the 5 words used in the exposure phase and participants in the unreliable condition knew on average 95% (*SD* = 8.4%) of the 10 words or object labels used in the exposure phase. There was no difference of vocabulary knowledge across these two groups, *t*(35) = 0.94, *p* = .34.

##### *Novel word trials*.

The novel word trials featured the two novel objects on the screen. The novel objects were the two unfamiliar animals introduced during the teaching phase. One was a plush of Kirby, a character in Nintendo games, a round pink creature with an oversized head. The other looked like a rat with bunny ears and a trunk. At the end of the experiment, parents who came into the lab were asked whether their child was familiar with either animal; all parents said no. The novel words were both bisyllabic and did not have any phonological neighbors in children’s lexicon (“danu” and “modi”).

##### *Familiar word trials*.

The familiar word trials featured two familiar objects not used during Phase 1 (orange, butterfly, spoon, duck, cat, boat, hat, fish). Pictures were yoked in pairs (i.e., the orange always appeared with the butterfly) and each pair appeared twice during the test phase (one time for each referent). The familiar words were chosen to be likely known by children of that age range. According to Wordbank (Frank et al., 2017), 89% of British 24-month-old children understand the familiar words we used during the test phase.

#### Criteria for Trial and Participant Exclusion.

Trials for which we have more than 35% of trackloss were rejected. Note that we first preregistered a more stringent criteria of 25%, yet given the difficulty in recruiting, we decided to keep trials with 35% of missing data instead of 25%. This change was amended in the original preregistration while data collection was ongoing. Keeping the original criteria does not change the pattern of results, although some of the effects reported below were only marginally significant as more participants were excluded with the 25% criteria, thus reducing statistical power. Participants that provided fewer than two test trials were excluded, as were participants that were not attentive during Phase 1 and Phase 2 (not looking at the screen). Note that in the present sample none of the children were excluded based on these criteria. Children received on average 12.47 trials (6.00 novel word trials) after applying the criteria for trial rejection.

#### Measurement and Analysis.

We measured the time course of children’s gaze toward the target picture (excluding looks away from the screen). Gaze position on each trial was recorded via an eye-tracker (SMI) with a 33-ms sample rate. We inspected the time course of eye movements from the onset of the first occurrence of the target word (“Look at the [target]”) until the end of the trial (4 s). We assessed familiar and novel word comprehension as a preference for the matching object similarly to previous studies using the same teaching and testing phases (Dautriche et al., 2015; Dautriche et al., 2018) and following other research demonstrating a preference for the matching object during novel word comprehension in this age range (e.g., Messenger & Fisher, 2018; Yuan & Fisher, 2009). Since we did not expect any learning difference between the specific novel words being tested (“danu” or “modi”), we compared participants’ behavior across conditions (reliable vs. unreliable) collapsing looking behavior for all test trials. We note that toddlers could display above-chance performance either because they learned both words, or because they learned only one word and inferred the other word during the test phase by relying on mutual exclusivity (e.g., Diesendruck & Markson, 2001; Golinkoff et al., 1992; Graham et al., 1998; Halberda, 2003; Markman, 1989; Xu et al., 2005). While our data do not disambiguate between these two possibilities, it does not undermine our conclusions as any observed difference between the two experimental conditions would reflect differences in word learning.

**Figure 2.**
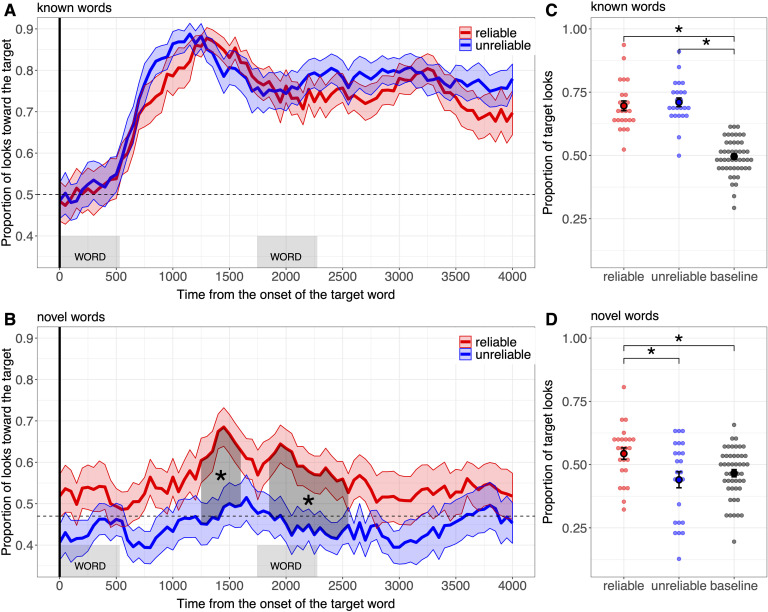
**Results of Experiment 1.A and B.** Proportion of looks toward the target picture during the familiar word trials (A) and the novel word trials (B), time-locked to the beginning of the first target word for children in the reliable condition (in red) and for children in the unreliable condition (in blue). The ribbon surrounding each curve represents the standard error of the mean obtained at each time bin for each word condition. The horizontal dashed lines represent the chance level: the average proportion of target looks during the baseline period. **C and D.** Overall proportion of looks toward the target in the reliable (red), in the unreliable (blue) conditions, and during the baseline period (black) for familiar word trials (C) and novel word trials (D). Individual points represent individual participant means. The error bars represent standard errors of the mean.

To test our hypothesis that children take into account speaker’s reliability when learning novel words we conducted two statistical tests. First, we ran a cluster-based permutation analysis (Maris & Oostenveld, 2007) as used previously in eyetracking studies (Dautriche et al., 2015; Ferguson et al., 2018; Hahn et al., 2015) on the proportion of target looks (downsampled in bins of 50 ms excluding away looks) across the whole trial duration. This type of analysis, originally developed for EEG data, does not require an a priori choice of a window of analysis (that could differ across reliability conditions, age, and word knowledge) and preserves the information available in the time-series. The cluster-based permutation analysis proceeds in two phases. First, we define clusters in the data: Temporally adjacent time-points that show statistically significant effects. For each time point, we compute a paired two-tailed *t* test comparing fixations across conditions (reliable vs. unreliable). All fixation proportions were transformed via the arcsin square function to better fit the assumptions of the *t* test. Adjacent time points with a *t* value greater than a predefined threshold (*t* = 2, as in Dautriche et al., 2015) are grouped together into clusters. We then define the size of each cluster as the sum of the *t* values from each of its constituent time points. Second, for each cluster, we assess the probability of observing a cluster of that size by chance through permutation. To do this we conduct 1,000 simulations where we randomly shuffle the relevant experimental conditions (i.e., reliable vs. unreliable) for each participant, while holding constant all other aspects of the data’s structure. For each permuted data set, we then identify clusters using exactly the same procedure as above, and reserve the largest of these clusters, eventually creating a distribution of largest clusters that were generated under this null (permuted) hypothesis. We then compare the clusters from the real (unpermuted) data to this null distribution. A real cluster shows a significant effect of condition if it is greater than 95% of the simulated clusters, implying a *p* value less than .05. Note that if no cluster is found in the original data set (no adjacent time points have a *t* value greater than the predefined threshold), the second step involving data permutation cannot be run (as the statistics calculated gives the likelihood of a cluster arising by chance, would such a cluster exists) and thus we simply report the result of the first step (i.e., no cluster found). To test word recognition specifically, we conduct two additional (not-preregistered) cluster-based analyses comparing the proportion of target looks in each condition to the chance level. Because pairs of picture stimuli may not be equally interesting to children, it is standard practice to compare the proportion of target looks in a postnaming time window to the proportion of target looks in a prenaming, or baseline, time window for each trial (Bergelson & Aslin, 2017; Swingley & Aslin, 2007). Thus, the chance level is determined from the average proportion of target looks before hearing any audio material (the baseline period corresponding to the first 2 s of the trial) across all participants in both conditions. Baseline target preference (see also Figure 2C and D) is 0.5 for known words (*SE* = 0.01) and 0.47 for novel words (*SE* = 0.01; significantly below the theoretical chance level of 0.5 according a one-tailed *t* test: *t* = −2.37, *p* = .02). The cluster-based permutation analysis proceeds similarly as the between-condition comparison except that at each time point we computed a one-tailed *t* test^2^ comparing the proportion of target look to chance (0.50 for familiar words and 0.47 for novel words).

Second, we compared the overall proportion of target looks, averaged across the whole trial duration (4 s), between the two conditions. We modeled the proportion of target looks in a mixed-model analysis (Bates & Sarkar, 2004) in R using the following model: PropTargetLook ∼ Condition + (1 | Participant). We added this more standard analysis to our preregistered cluster-based analysis as recommended by previous research (Delle Luche et al., 2015). We also analyzed how the proportion of target looks differed from chance where chance was the average proportion of looks during the baseline period for both the reliable and the unreliable condition. This was used as the intercept in the mixed-model analysis.

### Results

The cluster-based analysis revealed no difference in target-looking fixations between speaker conditions on the familiar word trials (no time window found in the between-condition cluster analysis; Figure 2A): Both groups of children fixated the target above chance (in the reliable condition: from 550 ms to 4,000 ms, *p* < .001; in the unreliable condition: from 600 ms to 4,000 ms, *p* < .001). In the novel word trials, however, children in the reliable group looked significantly more toward the target object than children in the unreliable group (from 1,250 ms to 1,600 ms after target word onset, *p* = .04 and from 1,850 ms to 2,550 ms *p* = .01 after target word onset; Figure 2B) with only children in the reliable group looking toward the target above-baseline preference (from 1,250 ms to 1,750 ms, *p* = .01; no time window found by the cluster analysis for the unreliable group).

The mixed-effect analysis conducted on the overall target looking time also show no difference between conditions for the familiar word trials (*β* = 0.02, *t* = 0.80, *p* = .43), with children in both conditions looking to the target object significantly above their baseline preference (reliable: *M* = 0.70, *SE* = 0.02, *β* = 0.19, *t* = 9.1, *p* < .001; unreliable: *M* = 0.70, *SE* = 0.02, *β* = 0.20, *t* = 10, *p* < .001). Yet, critically, for the novel word trials, children from the reliable group (*M* = 0.54, *SE* = 0.02) looked significantly more toward the target than children in the unreliable group (*M* = 0.44, *SE* = 0.03, *β* = 0.10, *t* = 3.38, *p* < .001, Cohen’s *d* = 0.78). While the reliable group looked at the target object significantly above their baseline preference (*β* = 0.08, *t* = 3.16, *p* < .001, Cohen’s *d* = 0.75), the unreliable group did not (*β* = −0.02, *t* = −0.98, *p* = .33).^3^

### Discussion

Toddlers selectively learned words depending on the speaker’s past accuracy: they learned novel words when taught by a speaker who previously labeled known objects correctly but not when the speaker used incorrect labels. Of course, it could also be that children in the unreliable condition performed at chance because of general confusion arising from observing labels being used incorrectly. However, children responded with high accuracy on familiar words in both conditions (i.e., even in the unreliable condition), suggesting that this was not the case.

This result thus replicates the general findings of selective trust research that a speaker’s accuracy modulates the word learning behavior of children (e.g., Corriveau et al., 2009; Koenig et al., 2004; ; Koenig & Harris, 2005; Pasquini et al., 2007) using an implicit measure of word recognition (see also Luchkina et al., 2018), in toddlers (see also Brooker & Poulin-Dubois, 2013; Luchkina et al., 2018).

However, the results of Experiment 1 do not directly speak to whether 2- to 3-year-old children are already equipped with a suite of cognitive processes for epistemic vigilance (Sperber et al., 2010). The existence of learning selectivity does not necessarily entail that children can evaluate the trustworthiness of the speaker to decide whether a word should be learned or not, as it could emerge through simple associative mechanisms: Children may ignore new information conveyed by an unreliable speaker, because this speaker is more associated with being incorrect in general. Even 14-month-olds selectively avoid learning from inaccurate/unconventional models (Buttelmann et al., 2013; Chow et al., 2008) pointing to the idea that such a strategy is a robust (and sensible) component of cultural learning, but crucially does not require learners to make inferences on others’ and their own knowledge states.

The next experiment provides the critical test for source monitoring in toddlers: Can children reflect on how they come to know the meaning of a word in order to update their lexicon? In particular, we tested whether children can reevaluate a word mapping they recently learned when later discovering that the speaker that taught them the word (the source of their knowledge) is unreliable.

## EXPERIMENT 2

### Method

#### Participants.

Fifty-one English-speaking children, ranging from 24 months 21 days to 36 months took part in this experiment (*n* = 25 in the reliable condition and *n* = 26 in the unreliable condition; reliable condition: *M* = 29*M*, 19*D*, *SD* = 93*D*, 10 boys; unreliable condition: *M* = 31*M*, 7*D*, *SD* = 87*D*, 17 boys). As in Experiment 1, we aimed to test at least 24 children in each group. 7 additional children were replaced because of fussiness during the experiment resulting in too many missing trials (*n* = 6; see exclusion criteria), technical issues (*n* = 1). Participants were recruited in nurseries around Edinburgh (*n* = 18) and in the lab (*n* = 33).

#### Procedure, Design, and Material.

**Figure 3.**
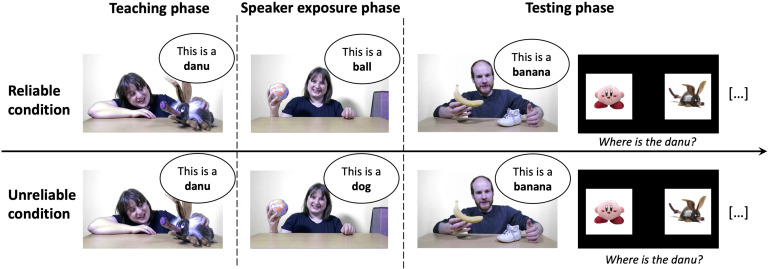
**Design of Experiment 2.** Experiment 2 consisted of the same three Experimental phases presented for Experiment 1, except that, critically, this time participants were first exposed to the teaching phase, where a speaker was teaching two novel words (“danu” and “modi”), before watching the video of the speaker exposure phase that revealed the (un)reliability of the speaker. The testing phase concluded the experiment and was identical to the one of Experiment 1.

The procedure and material were the same as in Experiment 1. The experiment was also composed of the three phases described in Experiment 1 but their order was different (see Figure 3): Participants were first exposed to the teaching phase, then to the speaker exposure phase before going into the test phase. Critically they first learned novel words before discovering whether the speaker that taught them these novel words was accurate or inaccurate.

#### Materials, Criteria for Trial and Participant Exclusion.

Same as in Experiment 1. Based on parental report (on the 43 questionnaires that could be collected, 19 from children assigned to the reliable condition and 24 from children assigned to the unreliable condition), participants in the reliable condition knew on average 97% (*SD* = 7.4%) of the five words used in the exposure phase and participants in the unreliable condition knew on average 97% (*SD* = 7.01%) of the 10 words or object labels used in the exposure phase. There was no difference in vocabulary knowledge on these word lists across groups, *t*(37) = 0.08,*p* = .94. Children received on average 11.39 trials (5.21 novel word trials) after applying the criteria for trial rejection.

#### Measurement and Analysis.

**Figure 4.**
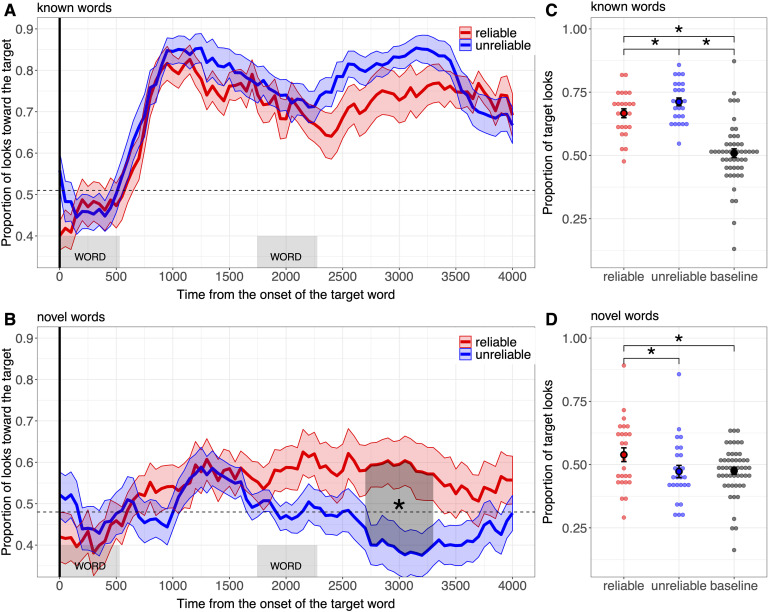
**Results of Experiment 2.A and B.** Proportion of looks toward the target picture during the familiar word trials (A) and the novel word trials (B), time-locked to the beginning of the first target word until the end of the trial for children in the reliable condition (in blue) and for children in the unreliable condition (in red). The ribbon surrounding each curve represents the standard error of the mean obtained at each time bin for each word condition. The horizontal dashed lines represent the chance level: the average proportion of target looks during the baseline period. **C and D.** Overall proportion of looks toward the target in the reliable (red), in the unreliable (blue) conditions and during the baseline period (black) for familiar word trials (C) and novel word trials (D). Individual points represent individual participant means. The error bars represent standard errors of the mean.

Same as in Experiment 1. In the cluster-based analysis comparing target looking behavior to chance, we define chance as the average proportion of looks toward the target during the baseline period as in Experiment 1 (familiar words: *M* = 0.51, *SE* = 0.02; novel words: *M* = 0.48, *SE* = 0.01; see also Figure 4). The baseline target preference was marginally different from 0.5 for the novel words according to one sample *t* test (*t* = −1.81, *p* = .07). In addition, we conducted another cluster-based permutation test comparing children’s target-fixation behavior between Experiments 1 and 2 for each condition (reliable; unreliable). Note that the mixed-effect model for novel words on the overall proportion of target looks came out to be singular. We, however, present the estimates of this model given that a Bayesian method forcing the random-effects variance-covariance matrix away from singularity gave similar results (see script online).

### Results

The findings of Experiment 2 were very similar to those of Experiment 1, even though children received evidence about speaker reliability *after* they had been taught the novel words (Figure 4).

Our cluster-based analysis revealed that the manipulation of reliability again did not affect children’s behavior on the familiar trials (*p*_*min*_ = .31; see Figure 4C), and children looked to the target object at above-baseline preference levels (in the reliable condition: from 750 ms to 4,000 ms, *p* < .001; in the unreliable condition: from 600 ms to 4,000 ms, *p* < .001). Second, and critically, speaker reliability did affect children’s behavior regarding the newly learned words. As in Experiment 1, there was a significant difference in performance between the reliable and the unreliable conditions during the novel word trials (from 2,750 ms to 3,350 ms after target word onset, *p* = .01) with children in the reliable group looking reliably more toward the target object than children in the unreliable group. As in Experiment 1, children in the reliable group looked toward the target above-baseline levels (from 1,900 ms to 2,100 ms, *p* = .04) but the unreliable group did not (*p*_*min*_ = .08). In addition, there was no difference between experiments in processing of the novel words: children’s fixation behavior in the reliable condition was comparable across Experiments 1 and 2 (no cluster found), similarly for children in the unreliable condition (no cluster found).

Our mixed-effect analysis revealed a preference for the target in the unreliable condition (*M* = 0.67, *SE* = 0.02) compared to the reliable condition (*M* = 0.67, *SE* = 0.02) during familiar trials (*β* = 0.05, *t* = 2.27, *p* = .01, see Figure 4D). Yet both children in the reliable group and in the unreliable group looked toward the target above-baseline levels (reliable: *β* = 0.15,*t* = 7.54,*p* < .001; unreliable: *β* = 0.21, *t* = 10.93, *p* < .001). Importantly, for the novel words, children in the reliable condition (*M* = 54, *SE* = 0.03) looked significantly more toward the target than children in the unreliable condition (*M* = 0.47, *SE* = 0.02, *β* = 0.07, *t* = 2.39, *p* = .01, Cohen’s *d* = 0.51). The reliable group showed target looks significantly above baseline preference (*β* = 0.05, *t* = 2.01, *p* = .04, Cohen’s *d* = 0.57) but not the unreliable group (*β* = −0.02, *t* = −0.69, *p* = .49).^4^ Similarly to the cluster-based analysis, our mixed-effect analysis revealed no difference between children’s fixation behavior across experiments for novel words: There was no main effect of experiment, *χ*^2^(2) = 0.88, *p* = .64, nor interaction between conditions and experiments, *χ*^2^(1) = 0.05, *p* = .82.^5^

### Discussion

Toddlers reevaluated a word mapping when they discovered that the source of their knowledge was unreliable. We suggest that the most likely explanation of this is that children continuously tracked the reliability of the source of their knowledge and used that information to reevaluate past information provided by this source. Such an interpretation relies on the assumption that toddlers successfully learned the novel word prior to reevaluating that knowledge when the speaker was subsequently shown to be unreliable. There is good reason to believe this is actually the case. First, studies using a similar teaching procedure show that children successfully learn novel words offered by a speaker they have no experience with (e.g., Dautriche et al., 2015; Swingley & Aslin, 2007; Waxman & Booth, 2002). Second, language has been highlighted as a domain in which children are thought to be particularly credulous simply because the risk of being misled while acquiring language is low. In normal circumstances speakers presumably do not have much interest to transmitting inaccurate lexical entries to children (except perhaps for a few teasing situations, a point we come back to later in discussing inferences about speaker motivations) if they want those children to share a common vocabulary with them (see also Sperber et al., 2010). Of course, this does not mean that children are fully credulous while building their vocabulary: 3- to 4-year-olds display better learning performance when a speaker is knowledgeable (Sabbagh & Baldwin, 2001) and as previous work (Koenig & Woodward, 2010) and our Experiment 1 have shown, children do not learn words from an unreliable speaker. Yet in the absence of any information about the speaker, children of this age range seem to generally accept the testimony of others as evidenced by their successful performance in word learning studies.

An unexpected observation from Experiment 2 is that the timing of the effect between the reliable and unreliable group appeared late, after participants heard the target word for the second time, while in Experiment 1 the effect emerged early, soon after participants first heard the target word. This may be because Experiment 2 presents a more challenging task for children, as it imposes a delay between the teaching phase and the testing phase of novel words. In such case the retrieval of the target object may be more effortful, and thus the effect delayed compared to Experiment 1. Whatever the exact nature of this process, the timing of word recognition in the two experiments may not be directly comparable. At any rate, our results show that children take speaker reliability into account independently of whether this reliability information comes before or after their word learning experience.

## GENERAL DISCUSSION

These experiments show that young children can reevaluate a word mapping that they recently learned, after subsequently discovering that the source of this knowledge was unreliable. This suggests that children are able to monitor the source of their knowledge and use that source monitoring for word learning from early in life, long before developing the ability to verbalize that information (see Gopnik & Graf, 1988; O’Neill & Gopnik, 1991; Taylor et al., 1994).

### Monitoring the Source of One’s Knowledge in the Wild

One immediate question is whether these experimental results generalize outside this laboratory context, such that young learners tag their lexical knowledge with information about how this knowledge has been acquired in real-world settings. Critically, in the experiments we reported, the word-mapping revision process depends on children’s capacity to encode the source of the word mapping (who taught them the word). This memory may be quite vivid in the present experimental context because children learned the words and witnessed the speaker’s reliability in a short sequence. More ecologically valid learning settings, however, would include multiple speakers, multiple words, and nonsequential learning events, all of which may make it harder for learners to track the source of their lexical knowledge. While the development of more sophisticated source-tracking abilities is an open question, there are two reasons to believe that children may be able to track the source of their lexical knowledge in the wild. First, multiple pieces of evidence suggest that word learning extends beyond label-referent associations: Children and adults track information about the contexts in which a word occurs. For instance, they can keep track of the semantic and contextual relations between words (e.g., that “shoe” is often uttered with “foot” and more frequently in a dressing-event than in a eating-event: Arias-Trejo & Plunkett, 2013; Bergelson & Aslin, 2017; Dautriche & Chemla, 2014; Perfetti & Hart, 2002; Wojcik & Saffran, 2013). And second, while young children were long thought to have very poor episodic memory, recent research suggest that these abilities had been underestimated by previous research (e.g., Király et al., 2018). Such an episodic trace may not only help the word learning process but also be an integral part of lexical entries, and as such be a naturally available source of information to learners.

In the present set of experiments we used the speaker’s identity as a convenient way to test whether children monitor and reflect upon the source of their lexical knowledge. Yet, speaker identity is clearly not the only type of source that children will need to monitor in order to learn words. For instance, word meanings can be taught directly by someone else, as in the present case, but they can also be inferred (as through mutual exclusivity inferences), or observed indirectly (as when learning from overheard speech). Thus, an important open question is whether children are also able to monitor these other sources of quality and reliability in terms of their lexical knowledge. Since young learners depend on communication with others to gain lexical knowledge, keeping track of who taught them what may be more critical, and more easily available, than monitoring which knowledge came directly versus from an inference. But monitoring of these latter processes may still be important, as children do rely on inferential processes, like judgments of mutual exclusivity or contrast (e.g., Clark, 1990; Markman, 1989), in order to acquire many words that are not ostensively taught. For instance, by 12 months, infants presented with a familiar and a novel object tend to choose the novel object when they hear “look at the dax” (e.g., Bion et al., 2013; Diesendruck & Markson, 2001; Golinkoff et al., 1992; Graham et al., 1998; Halberda, 2003; Xu et al., 2005). Inferential processes provide children with a powerful tool to learn a word within a single instance (Carey & Bartlett, 1978), yet it is unknown whether children recognize the inferential nature of a lexical entry acquired through such processes and weight it differently from a lexical entry acquired ostensively. Most tellingly, previous work suggests that identifying inference as a source of knowledge is particularly difficult for young children (O’Neill & Gopnik, 1991). It is thus open to question whether source monitoring extends beyond a speaker identity and applies to the many paths that a piece of knowledge can originate from.

A related question concerns the types of evaluations that toddlers can apply to the sources that they monitor. In the present set of experiments, as in previous studies (e.g., Corriveau et al., 2009; Koenig et al., 2004; Koenig & Harris, 2005; Koenig & Woodward, 2010; Luchkina et al., 2018), we have described our participants as being sensitive to the *reliability* of the speaker, defined as their tendency to conform to the conventions of a supposedly shared native language. But a speaker can be unreliable for many reasons: they could make a simple mistake, or be ignorant, or deceptive, or just joking, or maybe even from a different speech community. This highlights that an important aspect of source monitoring must be to infer why a source behaves in the way that they do. Such inferences are particularly important in the context of word learning, because the lexicon is a conventional system, in which two speakers can use two different labels for the same meaning, and both can be correct (cf. the difference between *soda* and *pop*, or *pants* versus *trousers*). Thus, children must learn to distinguish between the different motivations that sources may have for using words in seemingly unreliable ways. Research with preschoolers suggests that 5- to 6-year-old children put an increasing emphasis on speaker’s intention (e.g., being helpful or misleading) over the actual outcome they observe (e.g., Liu et al., 2013; Mascaro & Sperber, 2009; Vanderbilt et al., 2011), an ability that develops slowly over the preschool years (e.g., Vanderbilt et al., 2011) together with trait-reasoning development (e.g., Heyman & Gelman, 2000; Liu et al., 2007) and/or theory of mind development (Koenig & Harris, 2005). Yet, it is an open question whether younger children, whose sensitivity to inaccurate labeling develops early on (Koenig & Echols, 2003), can appraise the intention of unreliable speakers.

### Implications for Models of Word Learning

Our data indicate that children are not only able to monitor the source of their knowledge but they are able to use this information to update their lexicon. While children are skilled associative learners when acquiring the meaning of words (e.g., L. B. Smith, 2000), these results are difficult to explain under theories that rely on simple associative learning mechanisms, without important alterations that may no longer be in the spirit of associative learning. For instance, one could imagine that a lexical entry is associated to the *valence* of its source (e.g., Sumner et al., 2014). If the source has a negative valence (because it is atypical or inaccurate) this could affect the word as well. Yet it is unclear how children would retrospectively update a lexical entry as a function of its valence without involving some form of source monitoring. Rather, we suggest that revising a word meaning in this particular context is based on children’s self-reassessment of their knowledge. Word-mapping revision in this context implies that children are able to infer the correctness of the word mapping they have formed based on the informant’s accuracy. As such, this suggests that children are able to reflect on how they have come to know the meaning of a word, and use that information when constructing and updating their lexicon.

These results extend previous research suggesting that children can actively modulate their learning, not only by monitoring *what* they know and are interested in (e.g., Begus & Southgate, 2018; Goupil & Kouider, 2019; Lucca & Wilbourn, 2018), but also by monitoring *how* they know it, as we propose here. Yet, word learning models mainly focus on inferring word meaning based on observations (Siskind, 1996; Xu & Tenenbaum, 2007): if a learner hears “blicket” frequently while observing Dalmatians then they would infer that “blicket” means Dalmatian and not tree or dog. Much evidence, including the current study, suggests that learners are not only statistical accumulators but also display sensitivity to their own or others’ epistemic states to build their lexicon.

## CONCLUSION

In sum, our work shows that 2- to 3-year-old children can use a speaker’s accuracy to reevaluate a word’s meaning that was previously taught by that speaker. While pre-schoolers can reflect on how they have come to know the meaning of a word to guide word learning (Luchkina et al., 2020; Schütte et al., 2019; Scofield & Behrend, 2008), there was no evidence that younger children could monitor the source of their knowledge and use it to update their implicit beliefs. The present result suggests that these younger children track how they came to know the meaning of a word and use that information to update their lexicon. This research provides an important first indication that some form of self-reflective mechanism may play a key role in regulating knowledge-acquisition processes in the construction of the lexicon.

## ACKNOWLEDGMENTS

Many thanks to Jenny Chim, Rachel Kindellan, and Rebekah Oakley for data collection and to the actors of our stimuli, Jon Carr and Emma Healey. The research leading to these results received funding from the ESRC under the Future Research Leaders schemes (ES/N017404/1 and ES/N005635/1).

## FUNDING INFORMATION

ID, Economic and Social Research Council (http://dx.doi.org/10.13039/501100000269), Award ID: ES/N017404/1. HR, Economic and Social Research Council (http://dx.doi.org/10.13039/501100000269), Award ID: ES/N005635/1.

## AUTHOR CONTRIBUTIONS

ID: Conceptualization: Lead; Formal analysis: Lead; Methodology: Lead; Visualization: Lead; Writing - Original Draft: Equal; Writing - Review & Editing: Equal. LG: Formal analysis: Supporting; Writing - Original: Equal; Writing - Review & Editing: Equal. KS: Conceptualization: Supporting; Formal analysis: Supporting; Methodology: Supporting; Supervision: Equal; Visualization: Supporting; Writing - Original Draft: Equal; Writing - Review & Editing: Equal. HR: Conceptualization: Supporting; Formal analysis: Supporting; Methodology: Supporting; Supervision: Equal; Visualization: Supporting; Writing - Original Draft: Equal; Writing - Review & Editing: Equal.

## Notes

^1^ Note that this may be overly cautious depending on the theory of mind abilities of children of this age.^2^ Note that we used one-tailed *t* tests because our hypothesis was directional as we expected a higher-than-chance looking proportion when the word was recognized. Using two-tailed *t* test did not change the pattern of results; in particular, no cluster below the chance level passed the permutation test.^3^ It is to be noted that the comparison of prenaming and postnaming proportion of target looks may suffer from a temporal confound. One can imagine for instance that as more time passes, children become more bored with the objects overall, or shift their attention from familiar object to novel objects (e.g., Hunter & Ames, 1988). To deal with such a potential confound, we conducted a third analysis whereby, given paired pictures A and B, we calculated the fixation to picture A relative to B when A was the target, minus the fixation to A when A was the distractor (for each participant). A difference score above 0 would evidence word understanding. According to this approach participants learned the novel words in the reliable condition (difference score significantly above 0; *t* = 2.18, *p* = .03) but not in the unreliable condition (difference score not different from 0; *t* = −0.75; *p* = .45) and there was a significant difference between conditions, *F*(1, 41) = −2.04, *p* = .04. We note that using this measure requires that we have valid trials when A is a target and when A is a distractor. In the present case it was true for only 42 out of 48 participants, thus resulting in a loss of power compared to the pre- vs. postnaming analysis. Yet, despite reduced statistical power, this analysis replicates our main findings while dealing with the potential temporal confound of our baseline analysis.^4^ The mixed model used for the novel word analysis suffered from singularity. Using a Grubb test (Grubbs, 1950), we removed two outliers (one in the unreliable condition and one in the baseline; no other participant qualified as an outlier), this fixed the model’s singularity but did not change the pattern of results. Children in the reliable group looked significantly more toward the target than children in the unreliable group (*β* = 0.06, *t* = 2.87, *p* < .01) and above baseline levels (*β* = 0.04, *t* = 2.33, *p* = .02) contrary to the unreliable group (*β* = −0.02, *t* = −1.25, *p* = .21).^5^ Based on likelihood ratio tests on the following mixed-model PropTargetLook ∼ Condition * Experiment + (1 | Participant).
